# Characterization of Circulating Vesicles of Complicated and Uncomplicated Systemic Sclerosis Patients and Their Role in Vascular Dysfunction

**DOI:** 10.3390/ijms26062380

**Published:** 2025-03-07

**Authors:** Elena Grossini, Mattia Bellan, Sakthipriyan Venkatesan, Mohammad Mostafa Ola Pour, Marco Mennuni, Domenico D’Amario, Stefania Bruno, Daniela Ferrante, Daniela Capello, Pier Paolo Sainaghi, Mario Pirisi, Giuseppe Patti

**Affiliations:** 1 Laboratory of Physiology, Department of Translational Medicine, Università del Piemonte Orientale, 28100 Novara, Italy; sakthipriyan.venkatesan@uniupo.it (S.V.); 20046522@studenti.uniupo.it (M.M.O.P.); 2 Internal Medicine Unit, Department of Translational Medicine, Università del Piemonte Orientale, Azienda Ospedaliera Universitaria Maggiore della Carità, 28100 Novara, Italy; mattia.bellan@med.uniupo.it (M.B.); pierpaolo.sainaghi@med.uniupo.it (P.P.S.); mario.pirisi@med.uniupo.it (M.P.); 3 CAAD, Department of Translational Medicine, Università del Piemonte Orientale, Azienda Ospedaliera Universitaria Maggiore della Carità, 28100 Novara, Italy; 4 Cardiology Unit, Department of Translational Medicine, Università del Piemonte Orientale, Azienda Ospedaliera Universitaria Maggiore della Carità, 28100 Novara, Italy; marco.mennuni@med.uniupo.it (M.M.); domenico.damario@uniupo.it (D.D.); giuseppe.patti@med.uniupo.it (G.P.); 5 Laboratory of Translational Research, Department of Medical Sciences, University of Torino, 10126 Torino, Italy; stefania.bruno@unito.it; 6 Statistic Unit, Department of Translational Medicine, Università del Piemonte Orientale, 28100 Novara, Italy; daniela.ferrante@med.uniupo.it; 7 Laboratory of Clinical Biochemistry, Department of Translational Medicine, Università del Piemonte Orientale, 28100 Novara, Italy; daniela.capello@med.uniupo.it; 8 UPO Biobank, Department of Translational Medicine, Università del Piemonte Orientale, 28100 Novara, Italy

**Keywords:** biologic markers, mitochondria, oxidative stress, viability

## Abstract

Extracellular vesicles (EVs) could be involved in the onset of systemic sclerosis (SSc) through the modulation of vascular function. Anyway, available data are contradictory, and further investigation would be necessary to clarify this aspect. Here, we characterized circulating EVs isolated from SSc patients and evaluated their effects on human vascular endothelial cells (HUVECs) and smooth muscle cells. In EVs from 13 complicated and 27 uncomplicated SSc patients and five healthy controls (HCs), we analyzed the size, concentration, and surface marker expression. In addition, EVs were used to stimulate HUVECs, and we evaluated cell viability, mitochondrial membrane potential, and nitric oxide (NO) and mitochondrial reactive oxygen species (MitoROS) release. In smooth muscle cells, the effects of EVs on calcium movement were examined. The results showed that the EVs of SSc patients expressed markers of T-lymphocyte/platelet/endothelial cell origin and were larger and more concentrated than those from HCs. In addition, the EVs of SSc patients reduced cell viability and mitochondrial membrane potential and increased NO and MitoROS release in HUVECs and intracellular calcium in smooth muscle cells. In conclusion, we found a specific pattern for EVs isolated from SSc patients, which could have a pathogenic role through direct actions on endothelial and smooth muscle cells.

## 1. Introduction

Systemic sclerosis (SSc) is a rare autoimmune condition with clinical heterogeneity, associated with increased mortality and morbidity, as well as the impairment of patients’ quality of life [1,2,3,4,5,6]. Although the exact etiology is not known, it is widely accepted that environmental agents act as trigger factors in a genetically susceptible host to trigger inflammation and oxidative stress [7,8,9,10].

Regarding the pathogenesis of SSc, endothelial dysfunction and vasculopathy have been widely considered as the earliest events and hallmarks of disease [8,10]. In particular, the loss of endothelial function and the endothelial-to-mesenchymal transition (EndMT), likely related to the “inflammatory and pro-oxidant milieu”, could lead to evolution towards fibrosis and the onset of one of the most important causes of morbidity and mortality in patients with SSc, which is pulmonary arterial hypertension (PH) [11,12,13,14].

The molecular mechanism at the basis of endothelial dysfunction, EndMT, fibrosis, and transition towards PH would involve the release of cytokines and the activation of signaling pathways related to protein kinase B (Akt), phosfoinositide-3-kinase (PI3K), and mitogen-activated protein kinases (MEK1/2), like extracellular signal-regulated-kinase1/2 (ERK1/2) and adenosine 5′-monophosphate activated protein kinase (AMPK) [15,16,17].

In addition, the activated platelets and endothelial cells could secrete profibrotic mediators, inducing tumor growth factor 1 (TGF-1), serotonin, endothelin-1 (ET-1), and vesicles, which would further amplify the endothelial damage, lung fibrosis, and evolution of SSc into PH [18,19,20,21].

Also, the deepening of knowledge about the pathophysiology of SSc and its complications could have repercussions from a clinical point of view. Indeed, a considerable number of SSc patients have a poor prognosis, and identifying the disease in an oligo-symptomatic phase represents a challenge for its management [22]. This is particularly true for the screening of PH. To date, however, efforts to identify PH early in the course of the disease have been ineffective, and there is an urgent need to discover new discriminating biomarkers [23]. In this context, extracellular vesicles (EVs) could act as strong candidates. EVs are released from a variety of cells, such as platelets, endothelial cells, and leukocytes, and they act as important mediators of cellular cross-talk. Due to their involvement in inflammation, peroxidation, coagulation, and endothelial function, they could play a role in the pathogenesis and evolution of SSc and in the onset of PH [21,24,25,26,27].

Regarding this issue, it is important to note that high levels of circulating vesicles were found in SSc patients in comparison with healthy controls; moreover, an inverse relationship was observed between vesicles and microvascular complications, which suggests that they may also contribute to disease pathogenesis [24].

Those data would highlight the potential of those vesicles not only as a new field of investigation for deepening the knowledge about the physiopathology of the disease but also as early “biomarkers” in SSc. In this context, the use of EVs could help the early discrimination of patients with SSc and PH from those affected by interstitial lung disease alone or both conditions.

Although there is some information about the role of EVs in the pathogenesis of SSc and its complications, a better understanding of the nature of circulating EVs in patients with SSc and their role in the onset of vascular dysfunction would be mandatory.

For this reason, our primary endpoint was to characterize plasma EVs in SSc patients with and without PH and interstitial lung disease, as well as to examine their effects on human vascular endothelial cells (HUVECs) and smooth muscle cells. Experiments were also performed in the presence of inhibitors of the Akt/PI3K, AMPK, MEK1/2/ERK1/2, protein kinase A (PKA), and Ca^2+^-calmodulin dependent protein kinase II (CAMKII) pathways in order to examine the mechanisms of the above effects.

## 2. Results

The SSc patients and HCs did not differ in terms of sex and age. Differently, as shown in Table 1, complicated SSc patients were older than the uncomplicated ones. Furthermore, females represented the majority of the sample, with no differences between complicated and uncomplicated SSc patients.

### 2.1. EVs Characterization

We performed NanoSight and MACSPlex analyses in order to characterize the EVs isolated from the plasma of both SSc patients and HCs in terms of concentration, size, and surface marker expression.

**Figure 1:**
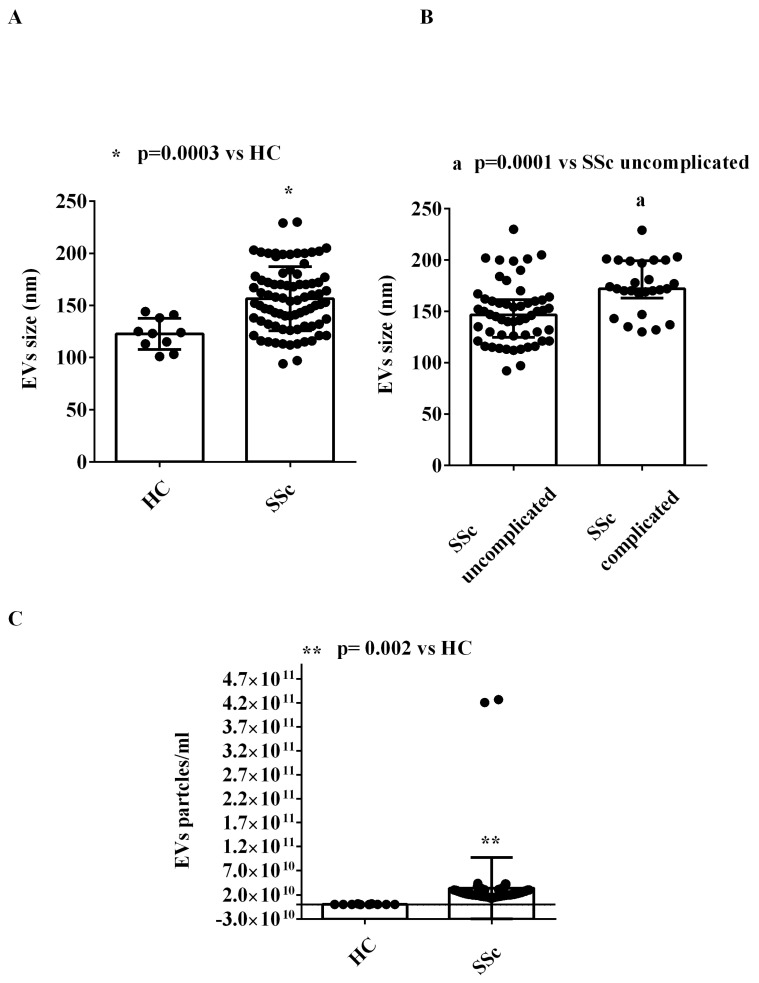
NanoSight analysis of extracellular vesicle sizes (**A**,**B**) and concentrations (**C**). The values are the median and range (min–max values) of measurements performed in duplicate. The Mann–Whitney test was used for the statistical analysis. A *p* value < 0.05 was considered for statistical significance. EVs = extracellular vesicles. HCs: healthy controls. SSc = systemic sclerosis.

As shown in Figure 1, the size of plasma EVs of SSc patients was greater than that of EVs isolated from HCs (Figure 1A). In addition, we found that EVs of complicated SSc patients had a larger size than those of uncomplicated SSc patients (Figure 1B). Moreover, the concentration of EVs isolated from the plasma of SSc patients was higher than that of SSc isolated from HCs (Figure 1C). The examples of NanoSight analysis are also shown in Figure S1.

We characterized the expression of 37 surface antigens in EVs isolated from the plasma of both SSc patients and HCs by using the MACSPlex Exosome kit.

**Figure 2:**
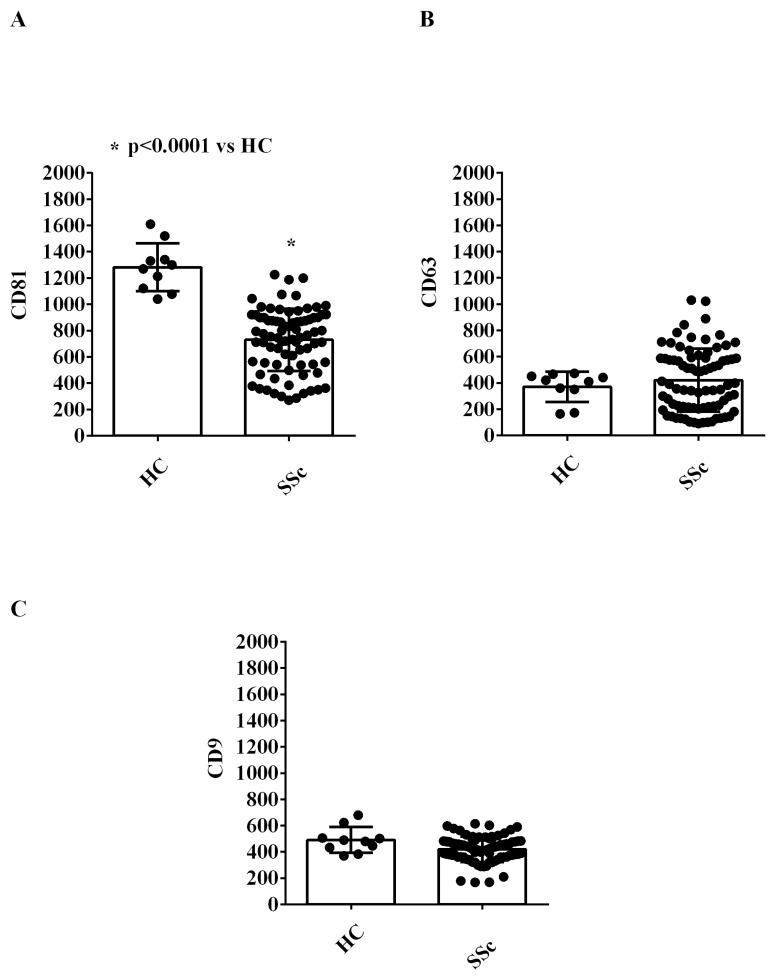
Results of MASPlex analysis. In (**A**–**C**), the expression of exosomal markers from the extracellular vesicles isolated from the plasma of SSc patients and healthy controls is shown. The results represent background-corrected CD9/CD63/CD81 MFI values normalized to the MFI of all detectable markers. Data are expressed as the median and range (min–max values) of measurements performed in duplicate. The Mann–Whitney test was used for the statistical analysis. A *p* value < 0.05 was considered for statistical significance. MFI: mean fluorescence intensity. HC: healthy control. SSc = systemic sclerosis.

**Figure 3:**
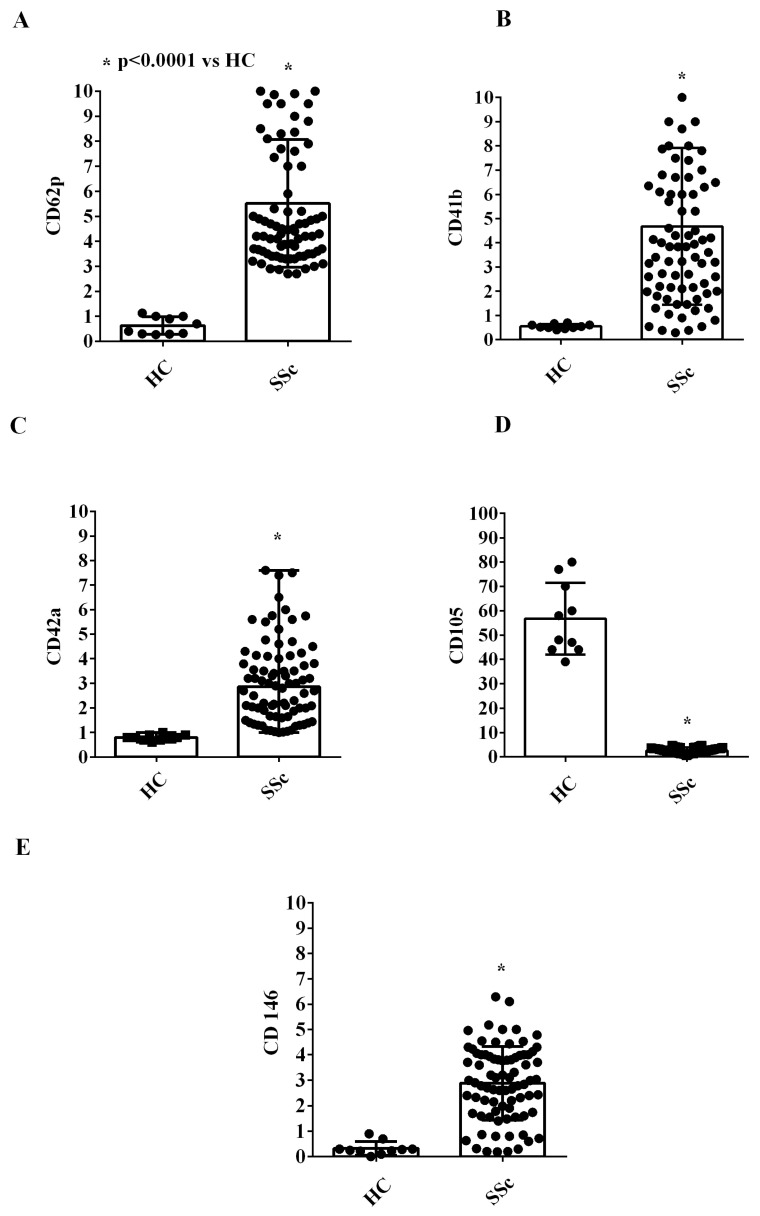
Expression of markers of platelet (**A**–**C**) and endothelial origin (**D**,**E**) from the extracellular vesicles isolated from the plasma of SSc patients and healthy controls. In (**A**–**E**), the fluorescence intensities of CD62p, CD41b, CD42a, CD105, and CD146, obtained via MACSPlex analysis, are shown. The intensity level of each marker was normalized to the MFI of all detectable markers and expressed as the median and range (min–max values) of measurements performed in duplicate. The Mann–Whitney test was used for the statistical analysis. A *p* value < 0.05 was considered for statistical significance. MFI: mean fluorescence intensity. HC: healthy control. SSc = systemic sclerosis.

It is noteworthy that EVs were positive for the typical exosome markers, CD9, CD63, and CD81 (Figure 2A–C). Moreover, EVs from all SSc patients were positive for platelet surface markers and endothelial surface markers (CD62p CD41b, CD42a, CD105 and CD146; Figure 3A–E), among which CD146 is considered as an endothelial marker of adhesion and is involved in inflammation and angiogenesis [28].

**Figure 4:**
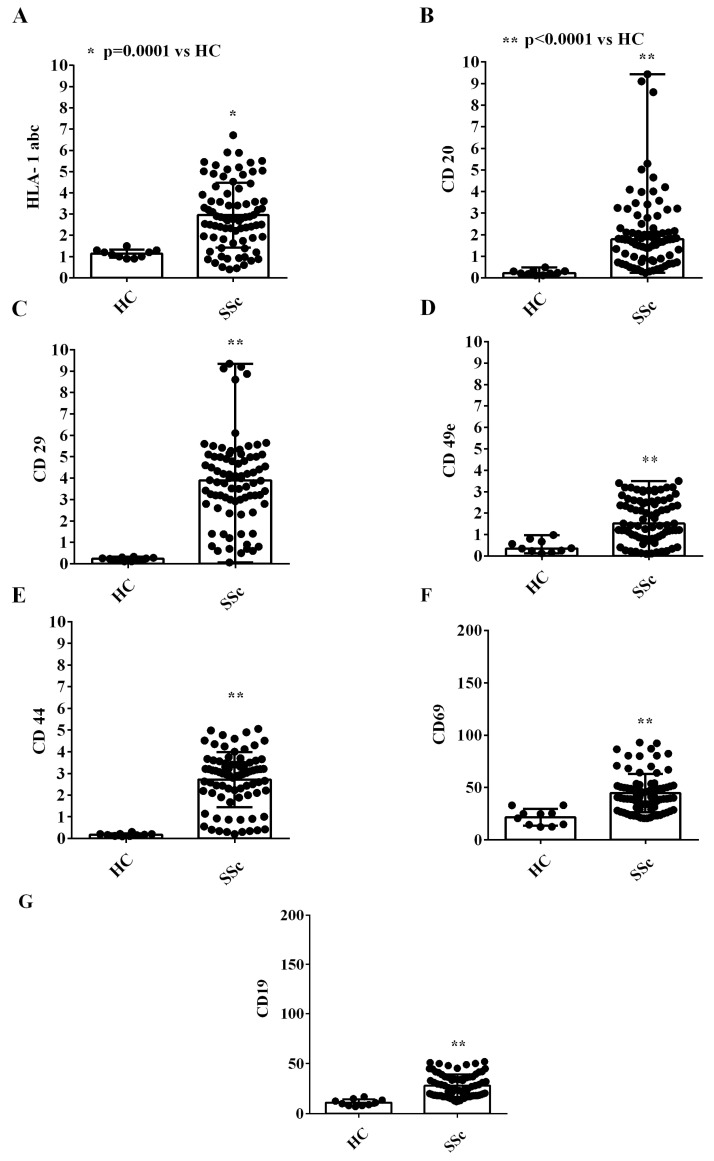
Expression of markers of inflammatory/hematopoietic origin from the extracellular vesicles isolated from the plasma of SSc patients and healthy controls. In (**A**–**G**), the fluorescence intensities of HLA-1 abc, CD20, CD29, CD49e, CD44, CD69, and CD19, obtained via MACSPlex analysis, are shown. The intensity level of each marker was normalized to the MFI of all detectable markers and expressed as the median and range (min–max values) of measurements performed in duplicate. The Mann–Whitney test was used for the statistical analysis. A *p* value < 0.05 was considered for statistical significance. MFI: mean fluorescence intensity. HC: healthy control. SSc = systemic sclerosis.

Also, we detected a higher expression of inflammatory/hematopoietic cell surface markers in EVs isolated from SSc patients vs. that found in EVs from HCs (HLA-1 abc, CD20, CD29, CD49e, CD44, CD69, and CD19; Figure 4). The comparison of the expression of all the above surface markers in EVs isolated from complicated and uncomplicated SSc patients did not show any statistically significant differences (Figures S1 and S2).

Regarding other markers, such as CD142, SSEA-4, CD31, CD24, CD1, CD11c, CD2, CD25, CD56, CD209, CD45, and CD40, we could only find some EVs from SSc patients to be positive and with very low values of measured fluorescence intensity.

### 2.2. Effects of EVs on HUVECs

**Figure 5:**
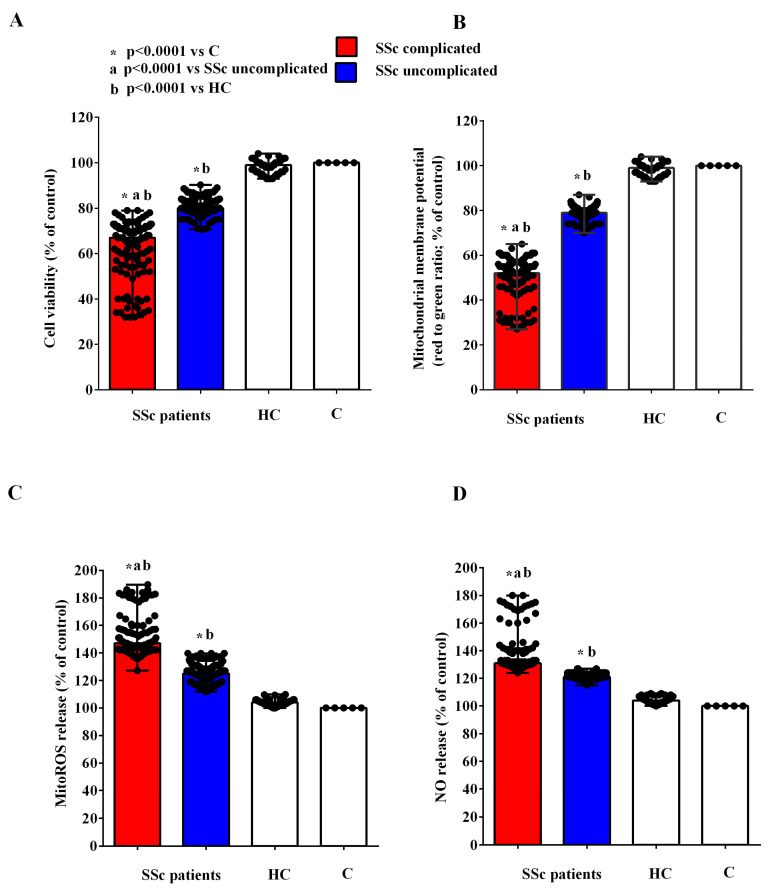
Effects of extracellular vesicles (50,000 EVs/cell) isolated from the plasma of SSc patients and healthy controls on cell viability (**A**), mitochondrial membrane potential (**B**), mitochondrial reactive oxygen species release (**C**), and nitric oxide release (**D**) in HUVECs. The results are the median and range (min–max values) of repeated experiments. The Mann–Whitney test was used for the statistical analysis. A *p* value < 0.05 was considered for statistical significance. MitoROS = mitochondrial reactive oxygen species release. NO = nitric oxide. C = untreated cells. HC = healthy control. SSc = systemic sclerosis.

After two hours of incubation, EVs obtained from the plasma of complicated and uncomplicated SSc patients were used to stimulate HUVECs (Figure S3). As shown in Figure 5A,B, in HUVECs stimulated with the EVs isolated from the plasma of SSc patients, cell viability and mitochondrial membrane potential were reduced in comparison with the findings obtained in HUVECs stimulated with the EVs isolated from HCs. In the meantime, the release of MitoROS and NO from HUVECs treated with the EVs of SSc patients was higher than that of HUVECs treated with the EVs from HCs (Figure 5C,D).

**Figure 6:**
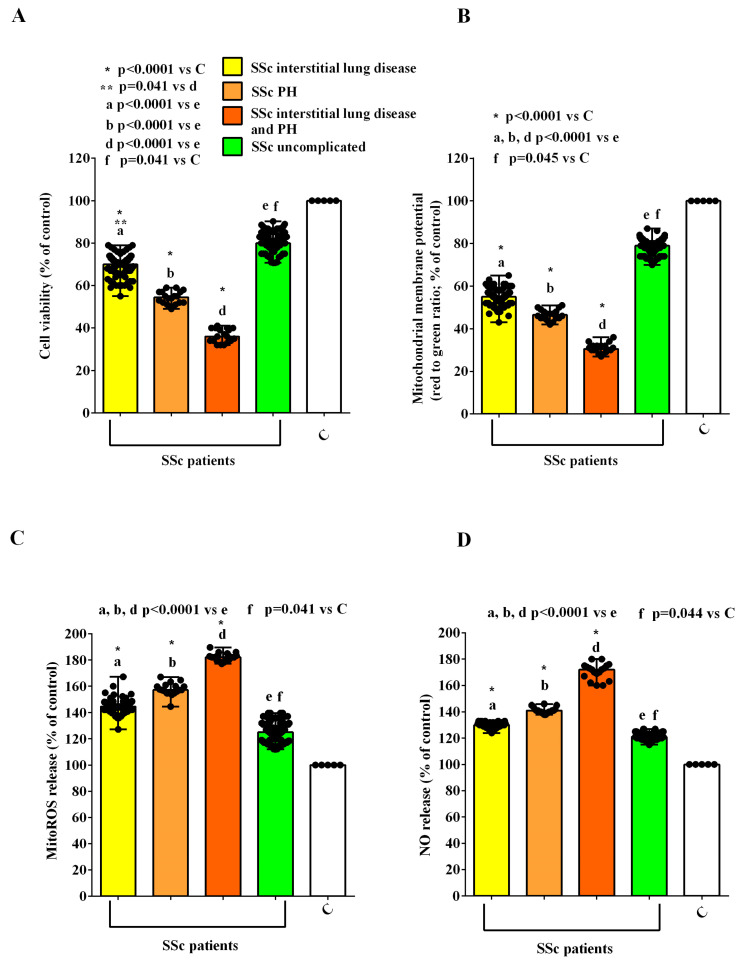
Effects of extracellular vesicles (50,000 EVs/cell) isolated from the plasma of SSc patients with interstitial lung disease alone, pulmonary hypertension alone, both interstitial lung disease and pulmonary hypertension, and uncomplicated patients on cell viability (**A**), mitochondrial membrane potential (**B**), mitochondrial reactive oxygen species release (**C**), and nitric oxide release (**D**) in HUVECs. The results are the median and range (min–max values) of repeated experiments. The Kruskal–Wallis test, followed by Dunn’s post hoc test, was used to perform multiple comparisons among various groups, applying Bonferroni correction. A *p* value < 0.05 was considered for statistical significance. MitoROS = mitochondrial reactive oxygen species release. NO = nitric oxide. C = untreated cells. PH = pulmonary hypertension. SSc = systemic sclerosis.

Moreover, we found stronger effects in HUVECs treated with EVs isolated from complicated vs. uncomplicated SSc patients. In particular, we observed that the reduction in cell viability and mitochondrial membrane potential was higher in HUVECs treated with the EVs isolated from SSc patients with interstitial lung disease alone, with PH alone, and with both complications (Figure 6A,B). Similarly, MitoROS and NO release were greater in HUVECs treated with the EVs isolated from complicated SSc patients than those found in HUVECs treated with the EVs isolated from the uncomplicated ones (Figure 6C,D).

**Figure 7:**
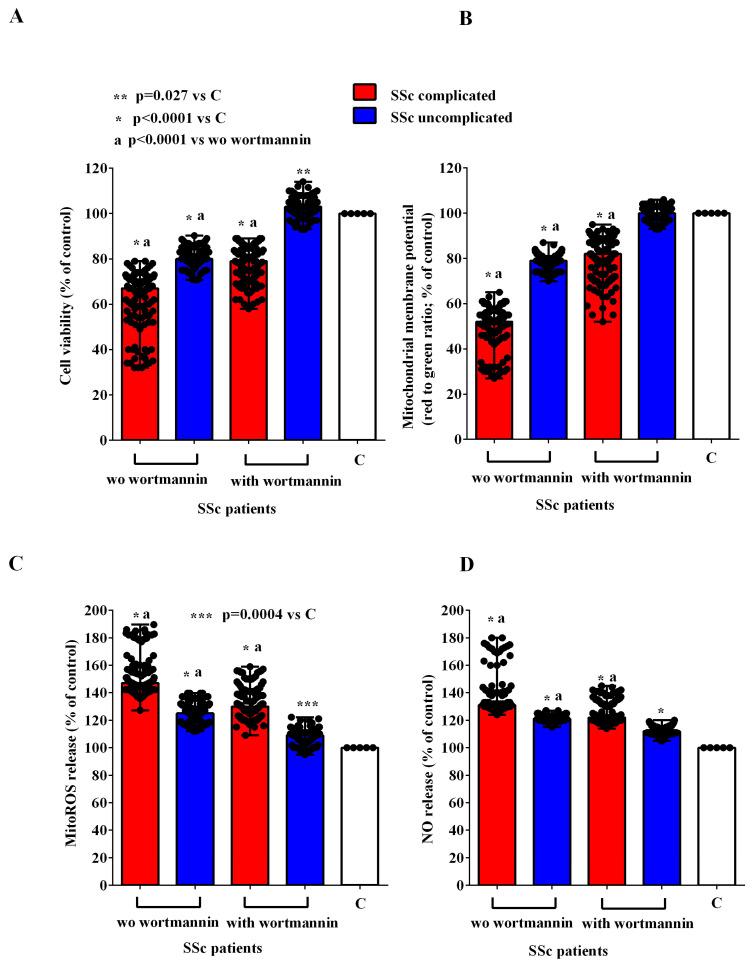
Effects of extracellular vesicles (50,000 EVs/cell) isolated from the plasma of SSc patients on cell viability (**A**), mitochondrial membrane potential (**B**), mitochondrial reactive oxygen species release (**C**), and nitric oxide release (**D**) in HUVECs in the absence and presence of wortmannin. The results are the median and range (min–max values) of repeated experiments. The Mann–Whitney test was used for the statistical analysis between the two groups (with the inhibitor vs. without the inhibitor). A *p* value < 0.05 was considered for statistical significance. MitoROS = mitochondrial reactive oxygen species release. NO = nitric oxide. C = untreated cells. SSc = systemic sclerosis.

**Figure 8:**
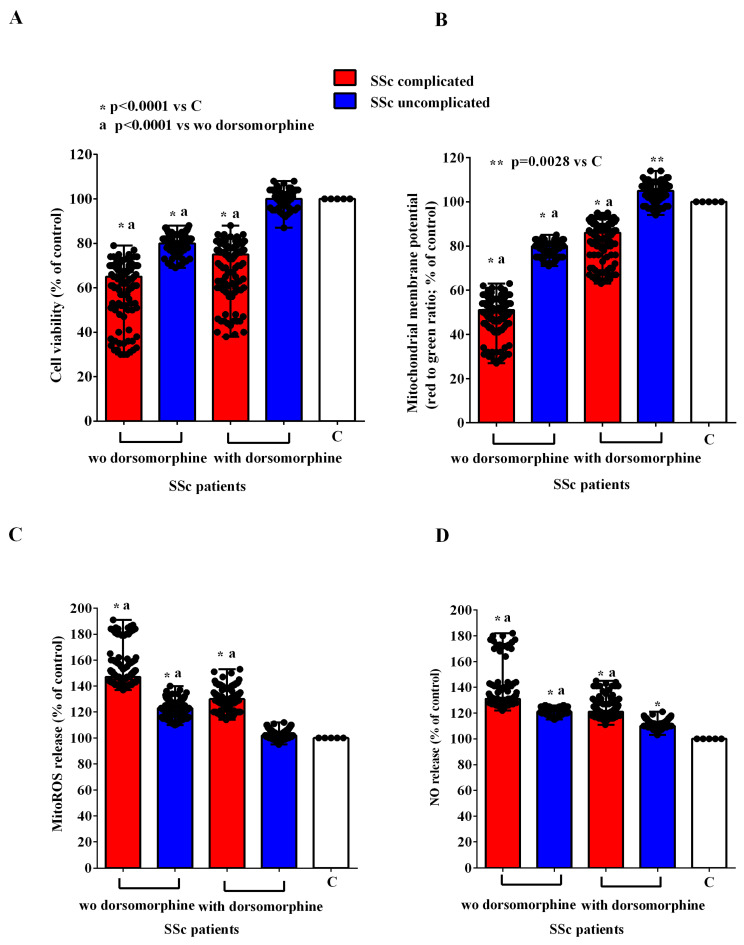
Effects of extracellular vesicles (50,000 EVs/cell) isolated from the plasma of SSc patients on cell viability (**A**), mitochondrial membrane potential (**B**), mitochondrial reactive oxygen species release (**C**), and nitric oxide release (**D**) in HUVECs in the absence and presence of dorsomorphine. The results are the median and range (min–max values) of repeated experiments. The Mann–Whitney test was used for the statistical analysis between the two groups (with the inhibitor vs. without the inhibitor). A *p* value < 0.05 was considered for statistical significance. MitoROS = mitochondrial reactive oxygen species release. NO = nitric oxide. C = untreated cells. SSc = systemic sclerosis.

**Figure 9:**
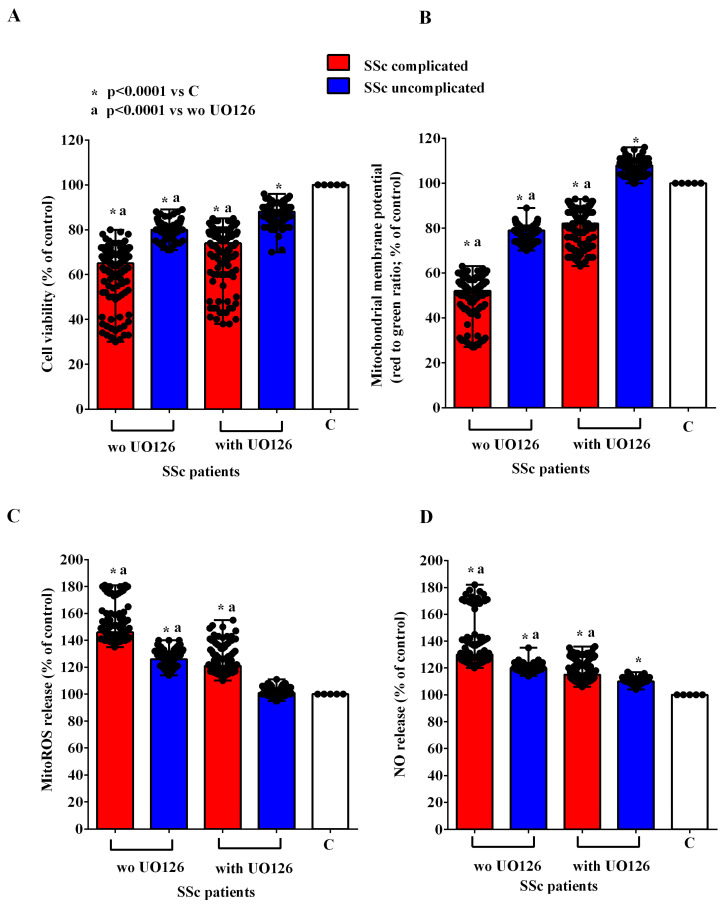
Effects of extracellular vesicles (50,000 EVs/cell) isolated from the plasma of SSc patients on cell viability (**A**), mitochondrial membrane potential (**B**), mitochondrial reactive oxygen species release (**C**), and nitric oxide release (**D**) in HUVECs in the absence and presence of UO126. The results are the median and range (min–max values) of repeated experiments. The Mann–Whitney test was used for the statistical analysis between the two groups (with the inhibitor vs. without the inhibitor). A *p* value < 0.05 was considered for statistical significance. MitoROS = mitochondrial reactive oxygen species release. NO = nitric oxide. C = untreated cells. SSc = systemic sclerosis.

**Figure 10:**
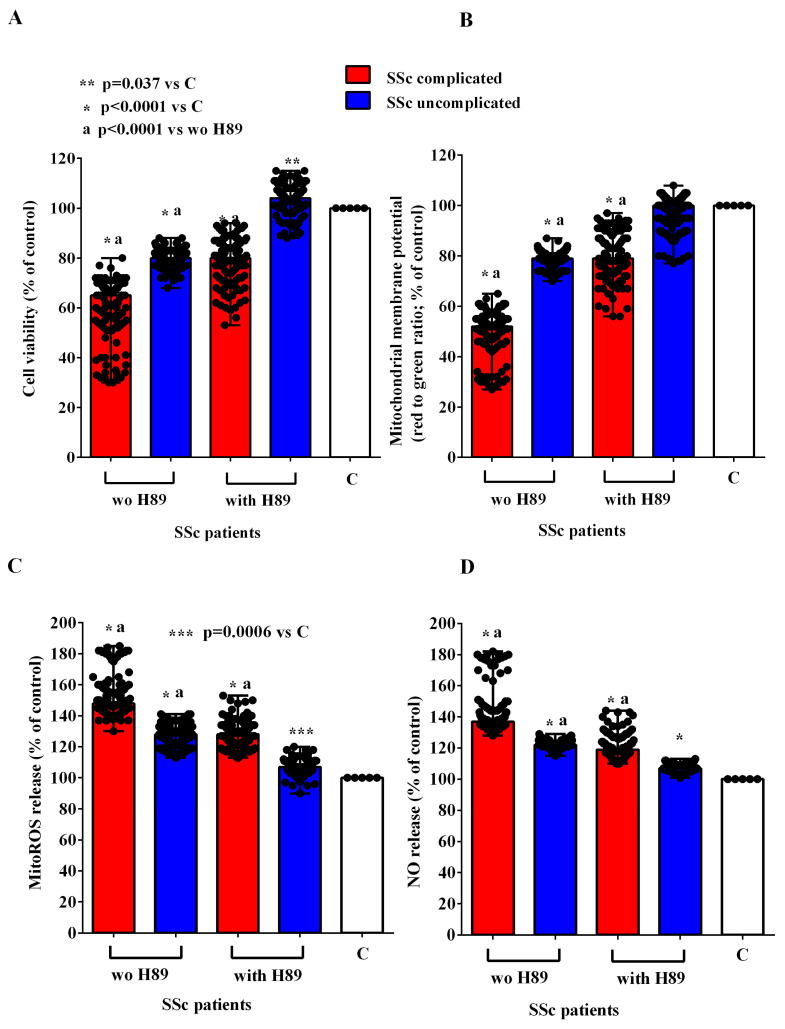
Effects of extracellular vesicles (50,000 EVs/cell) isolated from the plasma of SSc patients on cell viability (**A**), mitochondrial membrane potential (**B**), mitochondrial reactive oxygen species release (**C**), and nitric oxide release (**D**) in HUVECs in the absence and presence of H89. The results are the median and range (min–max values) of repeated experiments. The Mann–Whitney test was used for the statistical analysis between the two groups (with the inhibitor vs. without the inhibitor). A *p* value < 0.05 was considered for statistical significance. MitoROS = mitochondrial reactive oxygen species release. NO = nitric oxide. C = untreated cells. SSc = systemic sclerosis.

**Figure 11:**
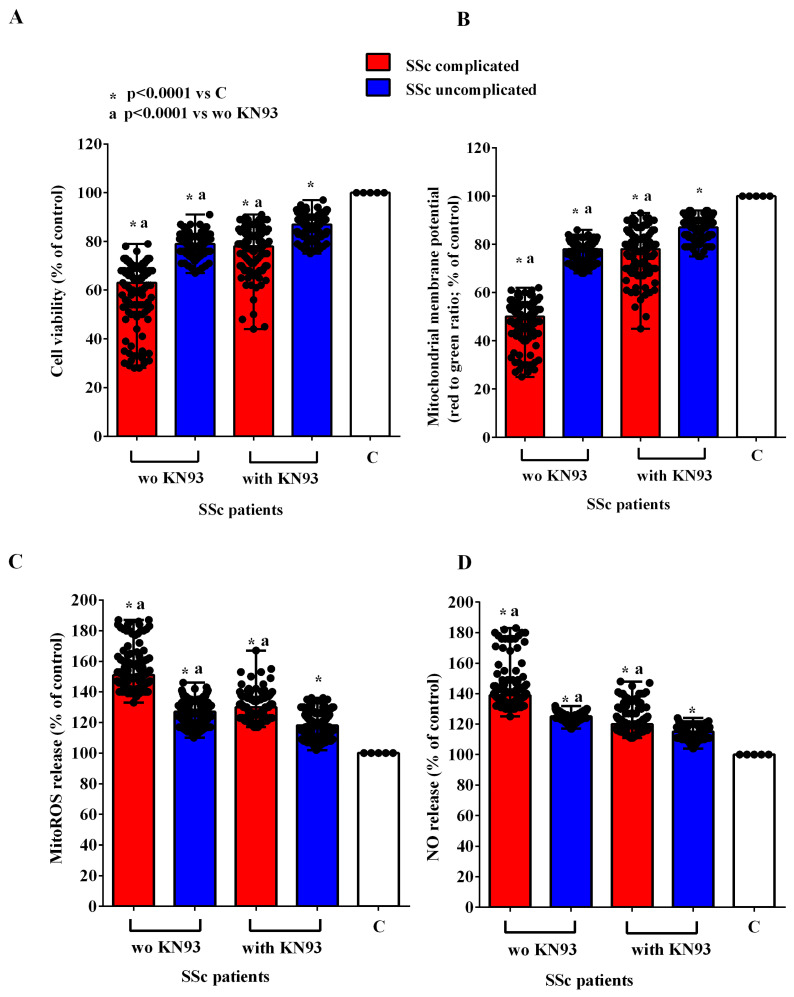
Effects of extracellular vesicles (50,000 EVs/cell) isolated from the plasma of SSc patients on cell viability (**A**), mitochondrial membrane potential (**B**), mitochondrial reactive oxygen species release (**C**), and nitric oxide release (**D**) in HUVECs in the absence and presence of KN93. The results are the median and range (min–max values) of repeated experiments. The Mann–Whitney test was used for the statistical analysis between the two groups (with the inhibitor vs. without the inhibitor). A *p* value < 0.05 was considered for statistical significance. MitoROS = mitochondrial reactive oxygen species release. NO = nitric oxide. C = untreated cells. SSc = systemic sclerosis.

In order to investigate the mechanisms of action involved in the effects of EVs of SSc patients in HUVECs, we conducted experiments in the presence and absence of inhibitors of the PI3K/Akt, AMPK, MEK1/2/ERK1/2, PKA, and CAMKII pathways, namely wortmannin, dorsomorphine, UO126, H89, and KN93, respectively. As reported in Figure 7, Figure 8, Figure 9, Figure 10 and Figure 11, all the inhibitors were able to counteract the effects of EVs of SSc patients. Hence, in the presence of wortmannin, dorsomorphine, UO126, H89, and KN93, the cell viability and mitochondrial membrane potential of HUVECs were greater than those found in the absence of the inhibitors. Also, the MitoROS and NO release were lower in HUVECs treated with the inhibitors vs. what found in HUVECs not treated with the inhibitors.

**Figure 12:**
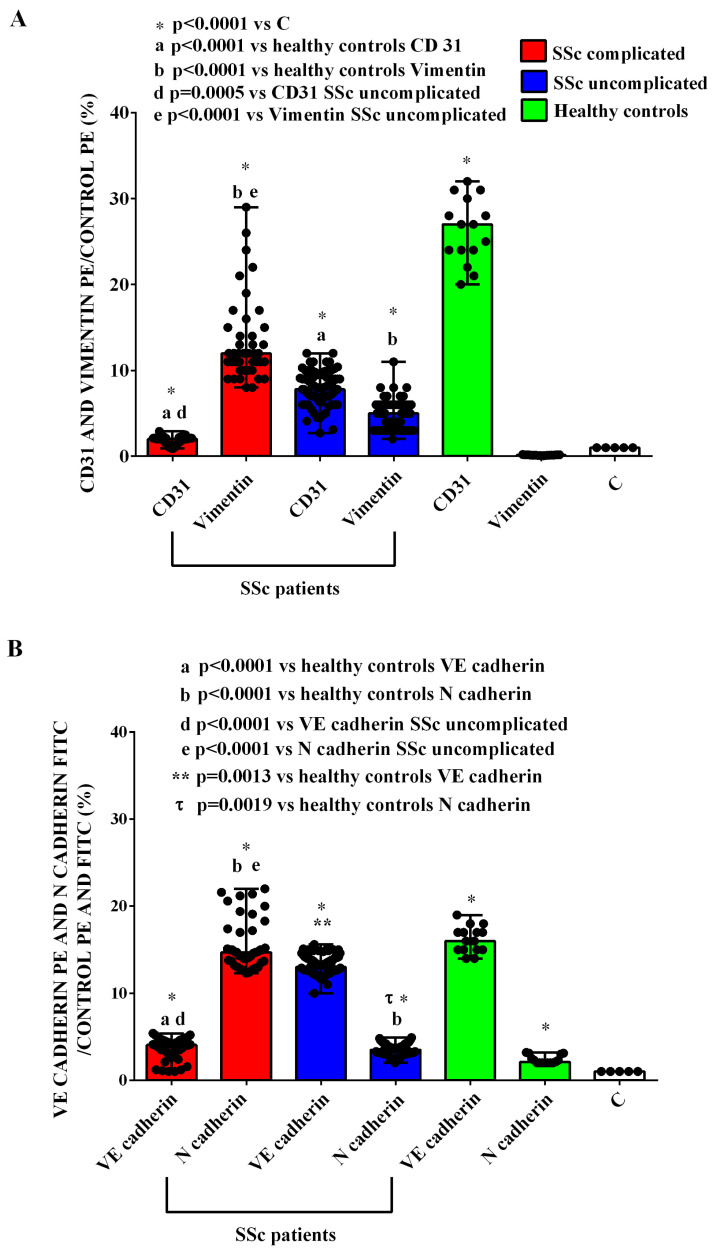
EndMt in HUVECs treated with extracellular vesicles (50,000 EVs/cell) isolated from SSc patients and healthy controls. In (**A**), CD31 expression and vimentin expression are shown. In (**B**), VE-cadherin and N-cadherin expressions are shown. The results are the median and range (min–max values) of experiments performed in triplicate. The Kruskal–Wallis test, followed by Dunn’s post hoc test, was used to perform multiple comparisons among various groups, applying Bonferroni correction. A *p* value < 0.05 was considered for statistical significance. C = untreated cells. SSc = systemic sclerosis.

The harmful effects elicited by the EVs of SSc patients in HUVECs were confirmed via the quantitative evaluation of EndMT, which was performed by analyzing the expression of CD31, VE-cadherin, vimentin, and N-cadherin in HUVECs in relation to the specific isotypic control (mouse FITC or PE IgG). As shown in Figure 12 and Table 2, the endothelial markers’ expression, namely CD31 and VE-cadherin, and the mesenchymal markers’ expression, namely vimentin and N-cadherin, were lower and higher, respectively, in HUVECs treated with the EVs isolated from SSc patients than those found in HUVECs treated with EVs isolated from HCs. As described in the Statistical Analysis Section and reported in the figures and table legends, the multiple comparisons among various groups were executed through the Kruskal–Wallis test, followed by Dunn’s post hoc test, applying Bonferroni correction.

In addition, HUVECs treated with the EVs from complicated SSc patients showed a reduced CD31 and VE-cadherin expression and an increased vimentin and N-cadherin expression in comparison with those found in HUVECs treated with EVs from uncomplicated SSc patients (Figure 12, Table 2).

### 2.3. Effects of EVs on C2C12

In C2C12 treated with EVs from SSc patients, we found a sudden increase in [Ca^2+^]c, which was similar in SSc with interstitial lung disease only and in uncomplicated patients. Instead, in SSc with PH and interstitial lung disease + PH, EVs caused a greater increase in [Ca^2+^]c (Figure S4).

It is important to note that in C2C12 treated with wortmannin, UO126, H89, and KN93, the effects of EVs on [Ca^2+^]c were reduced (Supplemental Table S1).

## 3. Discussion

The results of this study highlight a distinct pattern of circulating extracellular vesicles (EVs) in systemic sclerosis (SSc) patients, characterized by the expression of surface markers of platelet, inflammatory, and endothelial origin. Furthermore, we observed that these EVs may contribute to vascular dysfunction and hyperreactivity, which are considered as hallmarks of SSc, by damaging human vascular endothelial cells (HUVECs) and increasing intracellular calcium levels in smooth muscle cells. These effects were associated with the activation of intracellular pathways, including Akt/PI3K, AMPK, MEK1/2/ERK1/2, PKA, and CAMKII.

We focused on investigating the effects of EVs on HUVECs and C2C12 cells, as these cell types have emerged as potential indicators of endothelial integrity [10] and are implicated in the pathogenesis of SSc and its complications [7,29,30,31,32]. Supporting our findings, prior studies have reported differences in EV populations between SSc patients and healthy controls (HCs). Proteomic analyses have shown a higher expression of vascular markers in EVs from SSc patients [33]. Notably, Lammi et al. demonstrated that circulating EVs were increased in SSc–PH patients compared to those without PH, exhibiting higher levels of inflammatory cytokines, adhesion molecules, and CD44, which is a key endothelial junction marker involved in vascular permeability regulation [27]. Additionally, serum EVs from SSc patients have been found to stimulate extracellular matrix production, including fibronectin and collagen 1 [34]. Hypoxia-induced EVs, predominantly derived from platelets and erythrocytes, have also been shown to promote inflammation, oxidative stress, and endothelial NO synthase (eNOS) modulation in animal models [34].

Our study adds data to this body of evidence by demonstrating that SSc patients exhibit a distinct EV profile characterized by larger vesicle sizes and higher concentrations and express markers indicative of platelet, endothelial, and inflammatory cell origin. NanoSight and MACSplex analyses confirmed these findings, consistent with earlier observations by Guiducci et al. [25], who used flow cytometry to evaluate EV surface markers. In addition, our study expanded the analysis of EV surface markers via the use of an assay, which is designed for determining the concentrations of multiple soluble analytes in a single sample, indicating an inflammatory, endothelial, and platelet derivation of EVs. Since SSc is an inflammatory disease with vascular complications, we would consider the aforementioned antigen expression analysis of great importance in terms of the pathophysiology of the disease. It is important to note that although differences have been identified in the expression of antigens by the EVs of SSc patients compared to healthy controls, we could not detect any differences between complicated and uncomplicated SSc patients. This observation could be related to the small sample size, which compared 27 uncomplicated and 13 complicated SSc patients. In addition, although outside the scope of this study, the stage of disease progression and the duration of the disease may have also played a role in the failure to identify differences, specifically regarding the derivation of EVs between complicated and uncomplicated patients. Differently, the NanoSight analysis highlighted size differences between EVs from complicated and uncomplicated SSc patients.

Overall, the results obtained so far about EVs would suggest that they may represent pathophysiological markers of damage and the activation of the immune/inflammatory system in SSc. In addition, their characteristics, relating to the size and concentration, would also be related to the onset of complications of the disease, which could be associated with a different capability of transport of mediators and messengers. Regarding this issue, it is important to highlight the capacity of EVs isolated from SSc patients to enter HUVECs. This could represent the first step at the basis of the effects of EVs on endothelial cells, though they could induce damage.

The functional impact of these EVs on HUVECs and C2C12 cells suggests a pathogenic role in SSc, particularly in the onset of complications, such as pulmonary arterial hypertension (PH).

In order to perform the in vitro study on the above cell lines, we used 50,000 EVs/cell, since this concentration was similar to that of EVs quantified in the plasma of SSc patients and HCs. Furthermore, it was the one that, in preliminary experiments, had proven to be capable of reducing the cell viability of HUVECs in a similar manner, as performed by other concentrations amounting to 5000 EVs/cell and 500,000 EVs/cell. Regarding HUVECs and C2C12, we repeated the experiments on cell viability, mitochondrial membrane potential, mitoROS and NO release, and intracellular calcium levels at least five times and at least three times in the case of the experiments about EndMT and C2C12 in the presence of inhibitors. The aforementioned number of repetitions enhanced the statistical validity of the data. Regarding reproducibility across samples, it was notably high, as the results obtained from various assays and different samples were consistent. Furthermore, we added 1 μL of a solution containing EVs to each well. This solution was taken from a vortexed stock solution to ensure a homogeneous and uniform distribution of the EVs.

In HUVECs, the EVs from SSc patients reduced cell viability, decreased mitochondrial membrane potential, and increased mitochondrial reactive oxygen species (mitoROS) production. These effects were more pronounced in HUVECs treated with EVs from complicated SSc patients, with worsening responses observed in EVs derived from interstitial lung disease patients and those with interstitial lung disease and PH. Given the critical role of mitochondrial function in maintaining oxidative balance and regulating apoptosis [35,36], these findings underscore the detrimental impact of SSc and EVs on endothelial health. Mitochondrial dysfunction is also recognized as a contributor to SSc pathophysiology through innate immune activation [37].

EVs from SSc patients also altered NO release in HUVECs. Physiologically, endothelial cells produce small amounts of eNOS-derived NO, which promotes vasodilation through a cGMP-dependent signaling pathway [38]. Contrary to expectations, our study found increased NO release in HUVECs treated with SSc and EVs, particularly from complicated patients. This paradox may be explained by the shift in eNOS from a “coupled” to an “uncoupled” isoform under pathological conditions, producing greater NO quantities that convert to harmful peroxynitrites in oxidative environments [39,40,41]. Additionally, EV-mediated induction of the inducible NOS (iNOS) could further contribute to this phenomenon by altering NOS isoform expression.

Endothelial damage caused by EVs was further evidenced by the endothelial-to-mesenchymal transition (EndMT), as shown by the reduced expression of endothelial markers (CD31 and VE-cadherin) and increased mesenchymal markers (vimentin and N-cadherin). These effects were more pronounced with EVs from complicated SSc patients, aligning with the role of EndMT in endothelial dysfunction and fibrosis, particularly in SSc with PH [42]. Notably, agents like Iloprost, a prostacyclin analog used in SSc–PH treatment, have been shown to counteract EndMT by stabilizing adherens junctions [43].

In smooth muscle cells (C2C12), SSc and EVs induced rapid, transient increases in intracellular calcium levels ([Ca^2+^]c), particularly when using EVs from PH or interstitial lung disease + PH patients. This suggests a potential mechanism for EV-mediated vasoconstriction, contributing to PH pathogenesis in SSc.

To explore the pathways involved in the effects of EVs isolated from SSc patients on HUVECs and smooth muscle cells, which is an aspect not yet investigated, we conducted experiments by using inhibitors targeting Akt/PI3K, AMPK, MEK1/2/ERK1/2, PKA, and CAMKII. We chose to analyze the role of the aforementioned pathways since they have been implicated in the genesis of SSc and, more generally, in the regulation of the function of endothelial cells and of calcium movements [10,15,44,45,46].

The inhibition of these pathways reduced EV-induced effects in both HUVECs and C2C12 cells, confirming their involvement in SSc–EV-mediated vascular damage. These data added information about the mechanisms involved in the effects of the aforementioned EVs on the genesis of vascular dysfunction in SSc and are in agreement with what has been previously observed in a study where wortmannin and dorsomorphin were able to counteract the harmful effect of EVs isolated from HCV patients on HUVECs [47].

Collectively, our findings highlight the role of EVs in SSc-related vascular damage through mechanisms affecting endothelial and smooth muscle cells. SSc and EVs induce mitochondrial dysfunction, oxidative stress, altered NO production, and increase [Ca^2+^]c, potentially driving vasoconstriction and PH development. In addition to pathophysiological insights, our results suggest that EVs could serve as diagnostic and prognostic biomarkers in SSc. However, further studies with larger cohorts, especially with complicated SSc patients, are needed to validate these findings. Expanding our understanding about EV characteristics and their effects could pave the way for using EVs as biomarkers for early diagnosis and disease monitoring, addressing the current limitations of SSc biomarkers [22].

Finally, the therapeutic potential of EVs warrants exploration. Given their ability to modulate intercellular communication, EVs could serve as nano-delivery vehicles or biological carriers, offering innovative strategies for SSc treatment [48,49,50,51].

## 4. Materials and Methods

SSc patients were recruited from December 2022 to June 2023 at the Center for Translational Research on Autoimmune and Allergic Diseases (CAAD), Università del Piemonte Orientale, Novara, Italy.

According to our primary objective related to the role of plasma EVs in SSc patients with and without complications, the sample size was calculated starting from the literature’s data [27], considering a mean concentration of EVs of 1300/uL in SSc–PH patients vs. 560/uL in SSc patients without complications, and assuming a standard deviation of 500 MV/uL. On this basis, we calculated a sample size = 40 patients (with a prevalence of PH estimated at around 5–10% of the total patients recruited), which is sufficient to demonstrate a statistically significant difference in plasma EVs concentration between these two groups (power 80%, alpha error 0.05).

The study population included 35 females and 5 males with SSc, aged 63.9 ± 9.4 years. Among them, 27 had no complications, whereas 2 SSc patients had PH alone; 2 SSc patients had interstitial lung disease alone, and 9 SSc patients had both PH and interstitial lung disease.

This study was approved by the local Ethics Committee (CE058/23 (Option study), and all participants provided written informed consent. This study was performed per the ethical standards outlined in the 1964 Declaration of Helsinki and its subsequent amendments or comparable ethical standards.

A group of 5 healthy subjects of similar sex and age distribution (4 females and 1 male; age: 64 ± 2.2) served as healthy controls (HCs).

### 4.1. Plasma Sampling

In each SSc patient and HC, one or two blood samples were taken at 9 a.m. in fasting conditions by using BD Vacutainer tubes (sodium heparin as anticoagulant) at CAAD. Each sample was centrifuged for 10 min through a centrifuge model 5702 with rotor A-4-38 (Eppendorf SE; Hamburg, Germany) working at 3100 rpm and at 4 °C. The plasma was then aliquoted into 5 tubes and was used for the quantification of the most common laboratory analytes according to current standards of clinical practice and for EV isolation and the execution of the in vitro experiments. The plasma samples were stored at −80 °C at UPO Biobank, Novara, Italy. Plasma samples were always handled in pseudonymized conditions.

### 4.2. EVs Isolation

The isolation of EVs was performed through ultracentrifugation (Beckman Coulter Optima™ LE-80K; Beckman Coulter; Indianapolis, IN, USA) by using 2 mL of plasma samples diluted with phosphate-buffered saline (PBS) until a final volume of 4 mL of the tubes (Beckman Coulter) was reached. Thereafter, the tubes were positioned into the SW 60 Ti swinging-bucket rotor (Beckman Coulter), and the ultracentrifuge was set at 100,000× *g*, at 4 °C, and for 2 h, as previously performed [47,52,53,54]. Then, the supernatant was removed, and the pellet was re-suspended in 1 mL of fetal bovine serum (FBS; Euroclone S.P.A, Milan, Italy) in free Dulbecco’s Modified Eagle Medium (DMEM; Euroclone) and then stored at −80 °C.

### 4.3. EVs Characterization

We used NanoSight (NS300; Malvern Panalytical; Malvern, UK) equipped with the Nanoparticle Tracking Analysis (NTA) and NTA 3.2 Analytical Software Update to analyze isolated EVs, which have been diluted 1:200 in a 0.1 µm filtered physiological solution (sodium chloride 0.9%; Merck, Milan, Italy), as previously performed [55,56]. For each sample, a syringe pump flow of 30 was applied and three videos of 60 s each were recorded and analyzed, calculating an average number of EVs sizes and concentrations (particles/mL). The measurements were performed in duplicate.

### 4.4. MACSPlex Exosome Kit Analysis

The expression of 37 exosomal surface epitopes on EV surfaces was examined through the Human MACSPlex Exosome kit (Miltenyi Biotec; San Jose, CA, USA), as previously performed [55,56]. To do this, 1 × 10^9^ EVs (EV amount estimated based on NTA quantification analysis) were diluted in MACSPlex Buffer (MPB) until a final volume of 120 µL was obtained. After the addition of 15 µL of MACSPlex Exosome Capture beads, each sample was incubated overnight at 4 °C under gentle agitation and protected from light. Thereafter, EV–bead complexes were washed by adding 500 µL of MACSPlex buffer and centrifuged at 3000× *g* for 5 min at room temperature. A total of 500 µL of supernatant was then aspirated, and a mixture of anti-CD9, anti-CD63, and anti-CD81 (5 µL each) APC-conjugated antibodies was added to each sample. After 1 h of incubation under gentle agitation at room temperature, 500 µL of MACSPlex buffer was added, followed by centrifugation at 3000× *g* for 5 min. Afterwards, another washing was performed. Finally, after centrifugation, 350 µL of supernatant was removed, and the remaining part of the volume was used to resuspend the pellet. Flow cytometry analysis was performed using Cytoflex (Beckman Coulter). The median fluorescence intensity (MFI) of each capture bead subset was determined using the CytExpert software (version 2.0, Beckman Coulter). The background values of MACSPlex buffer and the isotype controls (recombinant engineered antibody (REA) or mouse IgG) were subtracted from each sample. The measurements were performed in duplicate. The values were then normalized to the mean MFI of the tetraspanins (CD9, CD63, and CD81).

### 4.5. HUVECs

HUVECs were purchased from ATCC (Manassas, VA, USA) (catalog no. CRL-1730™) and were maintained in DMEM (Euroclone) containing 2 mM L-glutamine (Euroclone), 1500 mg/L sodium bicarbonate (Euroclone) supplemented with 0.1 mg/mL heparin (Merck, Milan, Italy), 1% penicillin, 1% streptomycin, and 10% FBS (Euroclone).

HUVECs, cultured in 96-well plates at 10,000 cells/well, were stimulated for 24 h with EVs (50,000 EVs/cell diluted in PBS) from SSc patients and healthy controls. In particular, we examined the effects of EVs on cell viability (i.e., MTT assay), mitochondrial membrane potential (i.e., JC1 assay), nitric oxide (NO) release (i.e., Griess assay), and mitochondrial ROS release (MitoROS, i.e., MitoROS assay). The same evaluations were repeated in different pools of HUVECs treated with EVs in the presence or absence of the PI3k/Akt, MEK1/2/ERK1/2, AMPK, PKA, and CaMKII blockers, namely wortmannin (1 nM; Merck), UO126 (1 nM, Bio-Techne SRL, Milan Italy), dorsomorphine (1 nM; Merck), H89 (1 nM; Santa Cruz Biotechnologies, Dallas, TX, USA), and KN93 (1 nM; Merck). Simulations lasting 30 min were performed for all reagents [10,15,44,45,46]. The experiments on cell viability, mitochondrial membrane potential, and MitoROS and NO release were performed at least five times for each patients (or HC), whereas those regarding EndMT were executed in triplicate for each patients (or HC). All replicates were conducted by using different pools of HUVECs. This means that for each SSc patient or HC, we repeated the experiments at least five times in different pools of HUVECs by using the isolated EVs for each assay (MTT, JC1, MitoROS, and NO). In the case of the experiments about EndMT, we repeated the experiments three times in different pools of HUVECs by using the isolated EVs to examine the expression of CD31, vimentin, VE-cadherin, and N-cadherin.

### 4.6. Cell Viability Evaluation

In HUVECs, cell viability was investigated by using 1% 3-[4,5-dimethylthiazol-2-yl]-2,5-diphenyl tetrazolium bromide (MTT assay; Cayman Chemical, Ann Arbor, MI, USA), as previously performed [47,57,58,59]. We prepared a 10% MTT solution by dissolving 50 mg of the MTT reagent (3-(4,5-dimethylthiazol-2-yl)-2,5-diphenyl tetrazolium bromide) in 10 mL of PBS (pH 7.4; Euroclone) and kept it stored at 4 °C and protected from light. After the EV stimulation of HUVECs for 24 h, the media was removed, and 100 µL of the MTT solution diluted in high-glucose DMEM without phenol red (Euroclone), supplemented with 2 mM L-glutamine and 1% penicillin-streptomycin (P/S; Euroclone), was added to each well. Thereafter, the plate was incubated at 37 °C for 2 h. Thereafter, the supernatant was removed, and the formazan crystals were dissolved with 100 µL of dimethyl sulfoxide (DMSO; Merck). Finally, cell viability was measured by evaluating the absorbance through a spectrophotometer (VICTOR™ X Multilabel Plate Reader; PerkinElmer; Waltham, MA, USA) at a wavelength of 570 nm. Cell viability was calculated by setting untreated cells (control cells) as 100%.

### 4.7. Mitochondrial Membrane Potential Evaluation

We measured mitochondrial membrane potential (ΔψM) since it is an indicator of mitochondrial function and cell health. The medium of HUVECs stimulated with EVs (as performed for the MTT assay) was removed, and cells were incubated with the 5,51,6,61-tetrachloro-1,11,3,31tetraethylbenzimidazolyl carbocyanine iodide (JC-1) staining solution diluted in Assay Buffer 1X (Cayman Chemical) for 20 min at 37 °C, following the manufacturer’s instructions and as previously performed [47,57,58,59]. After incubation with EVs, HUVECs were washed twice with Assay Buffer 1X, and 100 µL/well of the solution was added for the final reading. Mitochondrial membrane potential was determined by measuring the red (excitation 535 nm/emission 595 nm) and green (excitation 485 nm/emission 535 nm) fluorescence through a spectrophotometer (VICTOR™ X Multilabel Plate Reader). Normalization of the data were executed against untreated cells (control cells).

### 4.8. MitoROS Release

We used the Mitochondrial ROS Detection Assay Kit (Cayman Chemical) to analyze mitoROS production in HUVECs, as previously performed [58,59]. To do this, the same experimental procedures used for the MTT and JC-1 determinations were adopted. After each treatment, the reactions were blocked by replacing the culture media with 120 µL of cell-based assay buffer. Then, the buffer was aspirated, and 100 µL of the Mitochondrial ROS Detection Reagent Staining Solution was added to each well and incubated at 37 °C, protected from light for 20 min. The staining solution was removed, and each well was washed three times with 120 µL of PBS. mitoROS release was analyzed by reading the excitation and emission wavelengths at 480 nm and 560 nm, respectively, through a spectrophotometer (VICTOR™ X Multilabel Plate Reader). Normalization of the data were executed against untreated cells (control cells).

### 4.9. NO Release

NO release in HUVECs was quantified using the Griess method (Promega, Milan, Italy) [47,57,58,59]. After the stimulation of HUVECs, as described for the previous assays, the supernatants were collected, and an equal volume of the Griess reagent was added to each sample according to the manufacturer’s instructions. The absorbance of each sample was read at 540 nm using a spectrometer (VICTOR™ X Multilabel Plate Reader). To quantify NO production, a standard curve was prepared, and the results were expressed as nitrites (μM). Normalization of the data were executed against untreated cells.

### 4.10. EndMT Analysis

To examine the EndMT, FACS analysis was executed on 10,000 HUVEC/well plated in 96-well plates and stimulated with EVs, as described for previous assays. After treatments, cells were detached and resuspended with 100 μL of the filtered physiological solution to make a final concentration of 10,000 cells/well. Then, 10 μg/mL of the antibodies anti-CD31-PE (Thermo Fisher Scientific, Rodano, Milan, Italy), anti-VE-cadherin-PE (Thermo Fisher Scientific), anti- N-cadherin-FITC (Thermo Fisher Scientific) and anti-vimentin-PE (Thermo Fisher Scientific) were added and incubated for 1 h at 4 °C in the dark. The analysis was performed by using the Attune™ NxT flow cytometer (Thermo Fisher Scientific). As specified above, as the control, the EVs were also incubated with a FITC mouse IgG isotype control or a PE mouse IgG isotype control (Thermo Fisher Scientific) [60,61].

### 4.11. Statistical Analysis

For each patient, the mean of the multiple measurements was considered for the analysis. All data are presented as the medians and range of different experiments performed on EVs, specifically regarding the characterization or different pools of HUVECs and smooth muscle cells. The differences between two or more groups were assessed through the Mann–Whitney test and the Kruskal–Wallis test, followed by Dunn’s post hoc test for multiple comparisons, with Bonferroni correction, respectively. Regarding categorical data, the statistical analysis was performed through the Fisher test. A value of *p* < 0.05 was considered statistically significant. Statistical analyses and graphs were executed by using GraphPad Prism version 9.0.0 (GraphPad Soft-ware; San Diego, CA, USA) and STATA v.17 (StataCorp. 2021 Statistical Software: Release 17; College Station, TX, USA).

## Figures and Tables

**Table 1:** Demographic, anthropometric and clinical characteristic of SSc patients.

	SSc Complicated (*n* = 13)	SSc Uncomplicated (*n* = 27)	*p* Value
Age (years)	68.5 ± 9.2	61.6 ± 8.7	=0.01
Females/males	12/13	23/27	>0.05
BMI (kg/m^2^)	22.6 ± 2.9	23.2 ± 4.8	>0.05
SSc phenotype (lcSSc vs. dcSSc)	lcSSc: 8/13 dcSSc: 5/13	lcSSc: 21/27 dcSSc: 6/27	>0.05
Overlap autoimmune diseases	2/13 (myositis, Sjogren)	3/27 (polymyositis, Sjogren, primary biliary cholangitis)	>0.05
WHO (1, 2, 3, 4)	1: 4/13 2:7/13 3:2/13	1: 19/27 2:8/27	WHO 1 = 0.038 WHO 2 > 0.05

Values are the means and standard deviations. BMI: body mass index; lcSSc: limited skin SSc; dcSSc: diffuse skin SSc; SSc: systemic sclerosis.

**Table 2:** Quantitative data about the endothelial-to-mesenchymal transition in HUVECs.

	C	HC	SSc Complicated	SSc Uncomplicated
CD31	1	27 (20–32) *	2 (0.9–2.9) *a	7.8 (2.7–12) *a
Vimentin	1	0.15 (0.07–0.21)	12 (8–29) *b	5 (2–11) *b
VE-cadherin	1	15 (12–19) *	4.05 (1–5.4) *d	13.1 (11.6–16) *d
N-cadherin	1	2.3 (2–4) *	14.7 (12.3–22) *e	3.5 (2–4.9) *e

Treated with EVs isolated from the plasma of SSc patients and HC. Median (minimum and maximum) percentage increase in CD31, Vimentin, VE-cadherin, and N-cadherin expressions in HUVECs treated with EVs isolated from the plasma of SSc patients and HC compared to untreated HUVECs (C). EVs: extracellular vesicles. HC: healthy control. * HC and SSc *p* value < 0.0001 vs. C; a CD31 SSc *p* value < 0.0001 vs. HC; b vimentin SSc *p* value < 0.0001 vs. HC; d VE-cadherin SSc complicated *p* value < 0.0001 vs. HC; VE-cadherin SSc uncomplicated *p* value *=* 0.0013 vs. HC; e N-cadherin SSc complicated *p* value < 0.0001 vs. HC; N-cadherin SSc uncomplicated *p* value *=* 0.0019 vs. HC. The Kruskal–Wallis test, followed by Dunn’s post hoc test, was used to perform multiple comparisons among various groups, applying Bonferroni correction.

## Data Availability

The data that support the findings of the present study are available from the corresponding author upon reasonable request.

## Supplementary Materials

The following supporting information can be downloaded at: https://www.mdpi.com/article/10.3390/ijms26062380/s1. References [62,63] are cited in supplementary materials.
