# Supplementary material for: Characterization of Circulating Vesicles of Complicated and Uncomplicated Systemic Sclerosis Patients and Their Role in Vascular Dysfunction
Source: Int J Mol Sci. 2025 Mar 7;26(6):2380. doi: 10.3390/ijms26062380 (PMC11942416; doi:10.3390/ijms26062380)
Supplement: Supplementary file 1 [file ijms-26-02380-s001.zip › ijms-3394300-supplementary.pdf]

Supplemental Materials and Methods

Incorporation of EVs in HUVEC

To study the capacity of EVs, obtained from complicated and uncomplicated SSc patients, to incorporate into HUVEC, we incubated for 2 h 50.000 EVs/cells. EVs had previously been labeled with Dil (Molecular Probes, ThermoFisher, Eugene, OR, USA) dye for 30 min at 37°C and washed by ultracentrifugation (100,000 g for 90 min). HUVEC were seeded in chamber slides (Corning, Glendale, AZ, USA; 10,000 cells/well) overnight. After 2 h incubation with labelled EVs, cells were washed with PBS and fixed with 2% paraformaldehyde for 30 min at 4° C. After, cells were incubated for 1 h with phalloidin FITC (Invitrogen, ThermoFisher, Eugene, OR, USA). Following PBS washes, Hoechst 33258 dye (Sigma Aldrich) was applied for nuclear staining. We evaluated the EVs incorporation by fluorescent microscopic analyses. Fluorescent microscopy analysis was conducted using a Zeiss AXIO Fluorescence Microscope (Carl Zeiss International, Oberkochen, Germany).

Measurement of [Ca^{2+}]c in C2C12 by Fura-2 Fluorescence

C2C12, which is an immortalized mice myoblast cell line that rapidly differentiate, forming contractile myotubes and producing characteristic muscle proteins and widely used for biomedical research, were purchased from ATCC (Manassas, VA, USA) (catalog CRL-1772) and maintained in DMEM (Euroclone) containing 2 mM L-glutamine (Euroclone), 1500 mg/L sodium bicarbonate (Euroclone) supplemented with 0.1 mg/mL heparin (Sigma), 1% penicillin, 1% streptomycin, and 10% FBS (Euroclone). As for HUVEC, also C2C12 cultured in 96-well plates at a density of 10000 cells/well, were stimulated with 50000 EVs diluted in PBS (Euroclone), per cell.

To measure [Ca^{2+}]c, C2C12 cells were grown to confluence, washed twice with sterile PBS (Euroclone), and incubated with 5 μM fura-2/acetoxymethyl (AM) ester (Sigma) in DMEM (Euroclone) containing 10% FBS and without phenol red (Euroclone) for 30 min in the dark. After additional washings with DMEM (Euroclone), the measurement was performed using a spectrometer (VICTOR™ X Multilabel Plate Reader) at an excitation wavelength of 340 nm and an emission wavelength of 510 nm. Fura-2/AM-loaded C2C12 cells were stimulated with EVs, as described for HUVEC. Also, the effects of EVs were compared with those of ATP (10 μM; Sigma). The experiments were performed at least five times for each patient (or HC) on different pools of C2C12.

Moreover, some experiments were performed in C2C12 treated with EVs in the presence of PI3k/Akt, MEK1/2/ERK1/2, PKA and CaMKII blockers, wortmannin (1 nM; Sigma), UO126 (1 nM; Bio-Techne SRL), H89 (1 nM; Santa Cruz Biotechnologies) and KN93 (1 nM; Sigma) 30 min stimulation. For each inhibitor, the experiments were performed in triplicate on different pools of C2C12.

The quantification of $[Ca^{2+}]c$ was obtained, as previously reported (30) by following this equation: (Ca^{2+}) = Kd ((R − Rmin)/(Rmax − R)). The Kd of Fura-2/AM for Ca^{2+} was considered as 224. Rmin and Rmax were the minimum and maximum values of fluorescence ratio obtained under Ca^{2+}-free (EGTA 0.1 M) or Ca^{2+}-saturated conditions, respectively [1,2].

Supplemental Figures

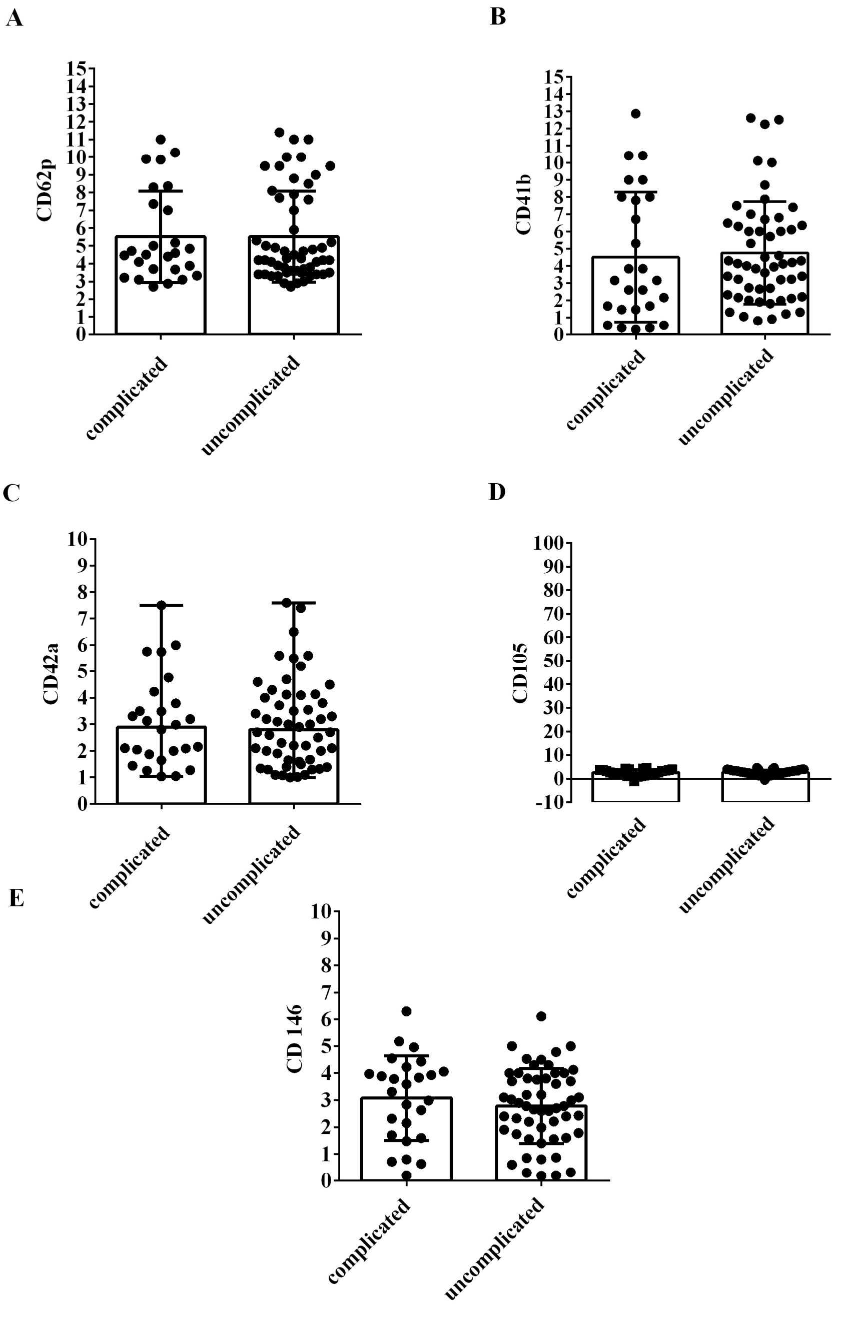

Figure S1. Expression of markers of inflammatory/hematopoietic origin by extracellular vesicles isolated from plasma of complicated and uncomplicated SSc patients. In A–G, fluorescence intensity of HLA-1 abc, CD20, CD29, CD49e, CD44, CD69 and CD19 obtained by MACSPlex analysis. The intensity level of each marker was normalized to the MFI of all detectable markers and expressed as median and range (min –max values) of measurements performed in duplicate. The Mann–Whitney test was used for the statistical analysis. A *p value <0.05* was considered for the statistical significance. MFI: mean fluorescence intensity.

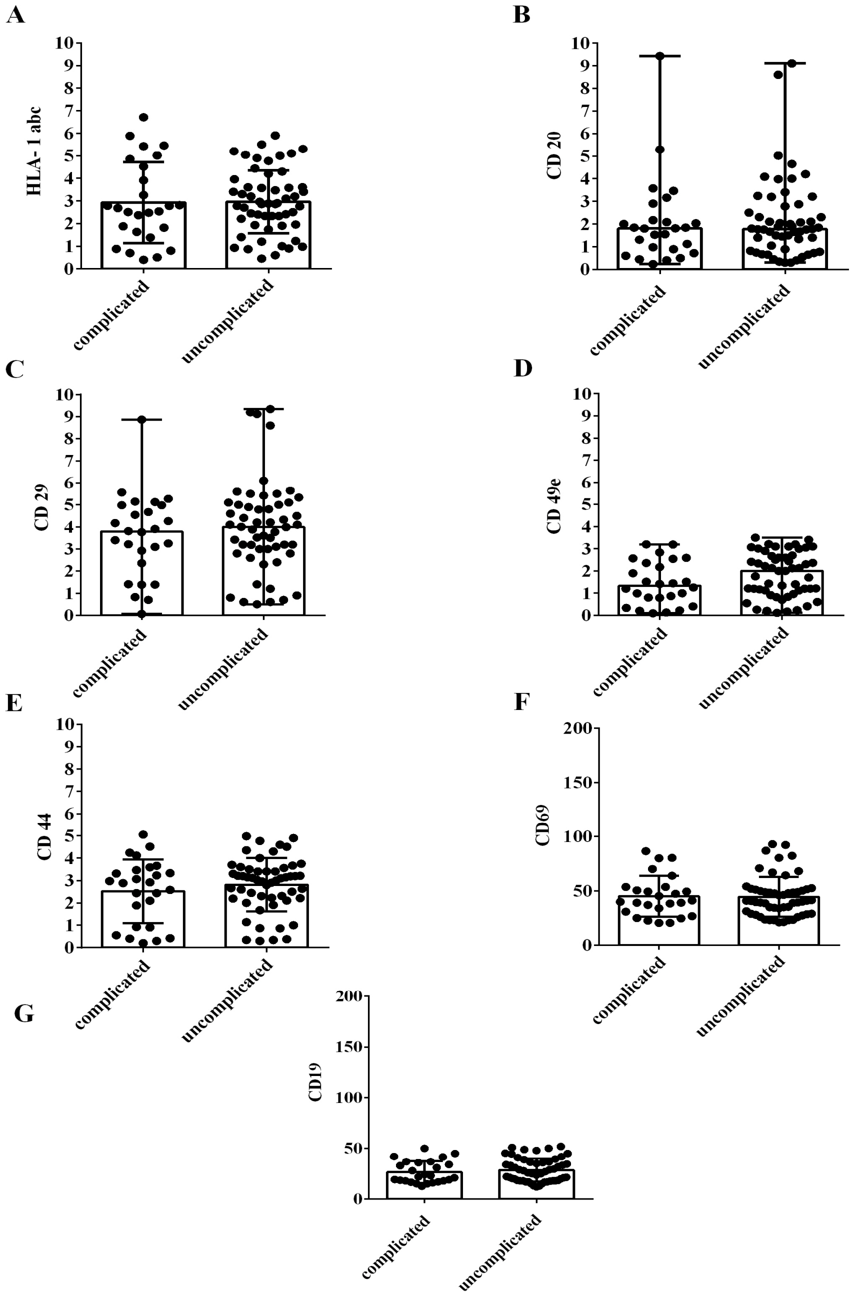

Figure S2. Expression of markers of inflammatory/hematopoietic origin by extracellular vesicles isolated from plasma of complicated and uncomplicated SSc patients. In A–G, fluorescence intensity of HLA-1 abc, CD20, CD29, CD49e, CD44, CD69 and CD19 obtained by MACSPlex analysis. The intensity level of each marker was normalized to the MFI of all detectable markers and expressed as median and range (min –max values) of measurements performed in duplicate. The Mann–Whitney test was used for the statistical analysis. A p value <0.05 was considered for the statistical significance. MFI: mean fluorescence intensity. SSc=systemic sclerosis.

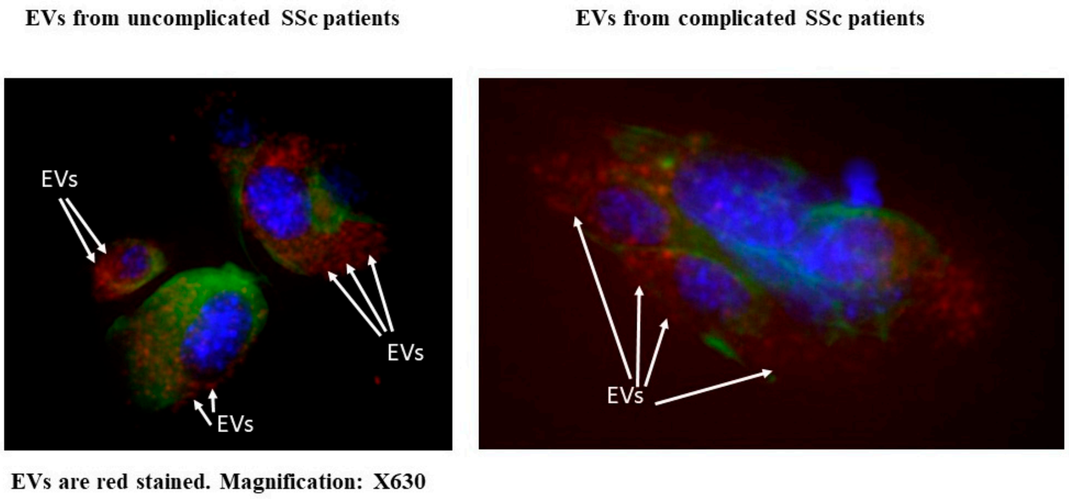

Figure S3. Entry of EVs into HUVEC

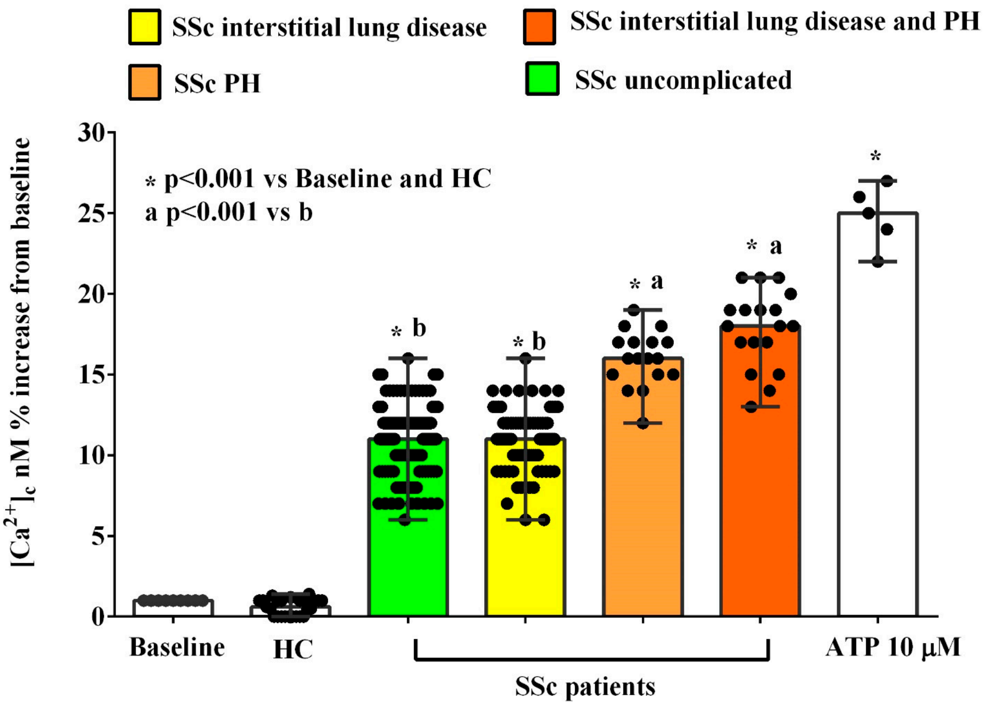

Figure S4. Effects of extracellular vesicles (50000 EVs /cell) isolated from plasma of SSc patients and healthy controls on intracellular calcium in C2C12. Results are median and range (min –max values) of measurements performed in triplicate. $[Ca^{2+}]c$: intracellular calcium. The Kruskal–Wallis test, followed by Dunn's post hoc test, was used to perform multiple comparisons among various groups, applying Bonferroni correction. A *p value <0.05* was considered for the statistical significance HC: healthy controls. PH: pulmonary hypertension. SSc= systemic sclerosis.

Supplemental Tables

Supplemental Table 1. Effects of extracellular vesicles isolated from plasma of SSc patients and HC on intracellular calcium in C2C12 with and without various blockers.

	SSc complicated	SSc uncomplicated	p
Baseline	1	1	
Without wortmannin	12 (9-19)*	11 (7-15)*	* <0.05
With wortmannin	6 (4-10)*	5 (2-12)*	*<0.05
Without UO126	12.5 (6-21)*	11 (6-15)*	*<0.05
With UO126	7.5 (5-11)*	6 (2-12)*	*<0.05
Without H89	12 (6-21)*	11 (7-15)*	* <0.05
With H89	7 (3-10)*	7 (3-13)*	*<0.05
Without KN93	12 (7-21)*	11 (6-15)*	*<0.05
With KN93	6 (3-8)*	4 (2-7)*	*<0.05

Results are median and range (min –max values) of measurements performed in triplicate. The Mann-Whitney test was used for the statistical analysis performed between SSc complicated and uncomplicated. A *p value <0.05* was considered for the statistical significance. * vs with blockers.

1. Grynkiewicz, G.; Poenie, M.; Tsien, RY. A new generation of Ca 2+ indicators with greatly improved fluorescence properties. *J Biol Chem*. **1985**, 260, 3440–3450.
2. Grossini, E.; Molinari, C.; Sigaudo, L.; Biella, M.; Mary, DA.; Vacca, G. Calcium handling in porcine coronary endothelial cells by gastrin-17. *J Mol Endocrinol*. **2013**, 50, 243–53.
